# Emotional Dysregulation in Adolescents: Implications for the Development of Severe Psychiatric Disorders, Substance Abuse, and Suicidal Ideation and Behaviors

**DOI:** 10.3390/brainsci10090591

**Published:** 2020-08-26

**Authors:** Domenico De Berardis, Michele Fornaro, Laura Orsolini, Antonio Ventriglio, Federica Vellante, Massimo Di Giannantonio

**Affiliations:** 1 NHS, Department of Mental Health, Psychiatric Service for Diagnosis and Treatment, Hospital “G. Mazzini”, ASL 4, 64100 Teramo, Italy; 2 Department of Psychiatry, Federico II University, 80138 Naples, Italy; dott.fornaro@gmail.com; 3 Psychopharmacology, Drug Misuse and Novel Psychoactive Substances Research Unit, School of Life and Medical Sciences, University of Hertfordshire, Hatfield AL109AB, UK; laura.orsolini01@gmail.com; 4 Unit of Clinical Psychiatry, Department of Clinical Neurosciences/DIMSC, Polytechnic University of Ancona, 60126 Ancona, Italy; 5 Department of Psychiatry, University of Foggia, 71121 Foggia, Italy; a.ventriglio@libero.it; 6 Department of Neurosciences and Imaging, University “G. D’Annunzio” Chieti, 66100 Chieti, Italy; federica.vellante@gmail.com (F.V.); digiannantonio@unich.it (M.D.G.)

**Keywords:** adolescence, emotions, regulation, anxiety, depression, suicidal ideation, addiction, alexithymia, prevention

## Abstract

Well-tuned emotional regulation is fundamental for human life and psychological well-being. Negative physiological emotions are counterbalanced by positive ones, and this equilibrium is the mainstay of human physiological affective states. However, this mechanism may sometimes become dysfunctional when negative emotions are not correctly counterbalanced, causing maladaptive behaviors, especially during adolescence. A very interesting review by Young et al. was recently published (*Brain Sci.* **2019**, *9(4)*, *76*) and stimulated us to reflect on this topic. The screening for emotional disturbances and dysregulation in adolescents must be included in all the preventive and interventional programs aimed to achieve both physical and psychological well-being of the population and early intervention should be provided in order to avoid progression toward clinically relevant psychiatric disorders in late adolescence and adulthood.

Adolescence, to date, is a high-risk period for developing several psychiatric disorders, and adolescents’ psychological well-being and mental health should be the first target of every prevention plan [1].

We know that adolescents may experience a wide range of emotions, often in an “all-or-nothing” way, experiencing them in extreme ways and quickly changing emotional states in response to environmental, parental, social, and internal factors [2]. This does not imply that such occurrence should always be considered a psychological disorder indicator. Still, it might be associated with a wide range of impulsive behaviors and reactions that are somewhat physiological for most, but within a certain set limits [3].

Emotional regulation can be described as how a person sustains, strengthens, or impedes his emotions according to his purposes or goals [4]. Emotional regulation is fundamental for human life, and well-being and emotions are usually in a balance between them. Negative physiological emotions are counterbalanced by positive ones, and this equilibrium is the mainstay of human physiological affective states [5]. However, this delicate and refined mechanism may sometimes become dysfunctional when negative emotions are not correctly counterbalanced [6]. This imbalance may cause maladaptive behaviors, especially during adolescence, a period where emotional states should be finely regulated [7].

A fascinating and scientifically sound review entitled “Positive and negative emotion regulation in adolescence: links to anxiety and depression” was recently published in *Brain Sciences* [8] and stimulated us to reflect on this topic. In this article, the authors reviewed the literature and correctly concluded that the disruptions in emotion regulation [8] are often associated with a higher probability of developing anxiety and depressive symptoms during adolescence [8]. Moreover, and this is one of the main findings of the review, these disturbances in emotion regulation are very predictive, rather than consequences, of forthcoming adult psychopathology. Interestingly, compromised abilities in emotional regulation may confer a further overall risk for all kinds of psychopathology, independently from its clinical appearance [8].

Some central questions raised by this excellent review could be “when the emotional regulation becomes dysfunctional, why, what will happen in this case, and what to do?”

Several studies have pointed out that emotional regulation may become dysfunctional when the regulation of negative and painful emotions is not counterbalanced adequately by positive and pleasant emotions, leading to an inability to tolerate intense, unpleasant, and persistent emotional states [9,10]. These persistent states are bothersome for the adolescent and may easily trigger the development of a “turmoil” of mixed anxious and depressive symptoms [10]. This outcome may be particularly dangerous in the adolescent, as he might try to regulate these undesired states and symptoms, for example, with substances use and abuse (especially cannabis or entactogen substances such as MDMA), addictive behaviors (Internet Addiction, Internet Gaming Disorder, Mobile-Phone Addiction, Online Gambling), and impulsive and risky conducts (including non-suicidal self-harm injuries) [11,12]. As a consequence, the emergence of suicidal ideation, together with the worsening of the psychiatric symptoms and hopelessness, rapidly becomes critical [13].

The apparent advantages of substance use and addictive behaviors are modifications in mood and personal experiences, relaxation and pleasure, and coping strategies against social anxiety, distress, tension, and, above all, persistent painful emotional states [14]. Still, these changes heavily reinforce the addiction, representing a temporary solution or a continuative coping strategy for handling developmental tasks (“escapism”) [12,15]. However, a vicious circle is triggered, and the more emotional dysregulation, the more reactive maladaptive strategies, and the more adolescent psychophysical health will be impacted with significant repercussions also in adulthood [16,17]. Moreover, suicide ideation and attempts in adolescents, even if often caused by psychiatric disorders, are reinforced by substance use together with psychosocial problems such as problems with individuals in the family and school environment, problems in interpersonal relationships, significant stress experiences, loneliness, competitiveness in school and home, and suicide history in the family [18,19]. Proper emotional regulation is the key to reduce the risk of the circumstances mentioned above, and preventive strategies must be put in place [20].

Not in all cases can the causative factors for emotional dysregulation be quickly and clearly found, and these should be accurately and empathically investigated in adolescence. Such factors include childhood traumas and maltreatment, sexual or physical abuse, neglect, learning disabilities, being a victim of bullying, parental distress, conflicts, severe parental psychiatric disorders, etc. [21,22]. In our opinion, when assessing an adolescent with unexplained emotional dysregulation, all those abovementioned issues should be considered.

In the review by Young et al. [8], the authors have correctly analyzed the pros and cons of several self-report tests that are easy to use as screening instruments for emotional regulation disturbances such as the Emotion Regulation Questionnaire (ERQ), the Difficulties in Emotion Regulation Scale (DERS), the Cognitive Emotion Regulations Questionnaire (CERQ), and the Fragebogen zur Ehrebung her Emotions regulation bei Kindern und Jugenlichen (FEEL-KJ).

In addition, behavioral assessment and peripheral psychophysiological correlates of emotion regulation were reviewed and can be conducted, but these procedures are more sophisticated, may require training for the assessor, and may be used further to evaluate ambiguous cases or persons positive to self-rating scales. Neuroimaging studies using functional Magnetic Resonance Imaging (fMRI) are very intriguing, but their use is, to date, limited to the clinical research.

In some cases, no antecedents can be found, and therefore, we believe that the possible presence of alexithymia should also be suspected and evaluated [23].

The alexithymic trait is often associated with impaired cognitive processing and regulation of emotions, and therefore, is likely to cause emotion regulation difficulties [24]; Taylor et al. have conceptualized alexithymia as a real “disorder of emotional regulation” [25]. The adolescents with significant levels of alexithymia manifest habitually higher subjective negative affects relative to autonomic activity irrespective of the intensity of environmental stresses [26]. The experimental observations have pointed out that persons with alexithymia have a greater tendency towards long-lasting negative emotions and show a propensity to suppress emotional expression to cope with these unpleasant and insupportable emotions [27]. The presence of alexithymia makes the adolescent more vulnerable, especially if the vulnerability has a genetic basis and may, per se, trigger anxiety, depression, substance abuse, and suicide ideation [28]. Thus, the early recognition of alexithymic traits should be implemented and the use of treatments that specifically focus on enhancing emotion must be administered [29,30].

Concerning neurobiological findings, the review by Young et al. [8] has demonstrated altered activation and connectivity in the amygdala and across regions of the prefrontal cortex to explain disrupted emotional regulation. These regions are implicated quite the same in alexithymia, suicidal ideation, and addiction, but these findings need further confirmations [31].

Concerning interventions, following Young et al. [8], we recommend the early administration of the Cognitive Behavioral Therapy (CBT) that underlines cognitive restructuring and encourages the use of reappraisal [32], and, as a second line of treatment, the so-called ‘third wave’ psychotherapies (i.e., mindfulness-based cognitive therapy, dialectical behavioral therapy) that converge on acceptance and decentering to regulate emotions [33]. There are also useful emotional-centered psychotherapies (such as Contextual Emotion Regulation Therapy and Emotion Regulation Therapy) that may be of particular importance, even before starting CBT or other ‘third wave’ psychotherapies, to restore an “emotional background” before starting structured psychotherapy [34]. The Positive Affect Treatment seems very promising and may also be employed in persons with alexithymic traits [35]. However, we believe that in severe cases, a concurrent pharmacotherapy should be considered based on the clinical picture and the neurobiological findings [36].

In conclusion, there is a need to detect early and evaluate dysfunctional and maladaptive emotional regulation in adolescents to prevent the development of psychiatric sequelae in adulthood. The screening for emotional regulation disturbances and alexithymia is relatively easy using validated self-report scales and can be further supported by behavioral and psychophysiological evaluations when available. The neural basis of emotional dysregulation is intriguing (such as reduced amygdala activation, and increased inverse ventrolateral prefrontal cortex–amygdala connectivity) [8], but further studies are needed. The risk of developing anxious and depressive symptoms in persons with unrecognized and untreated emotional dysregulation is high, and this is often further complicated by substance abuse, addictive behaviors, and suicidal ideation and attempts, generating a vicious circle that will be hard to break [37,38].

Thus, in our opinion and in accordance with the results of the review by Young et al. [8], the screening for emotional disturbances and dysregulation in adolescents must be included in all the preventive and interventional programs aimed to achieve both physical and psychological well-being of the population as it is “better safe than sorry”. Moreover, early intervention should be provided in order to avoid progression toward clinically relevant psychiatric disorders in late adolescence and adulthood.
